# Locally Aggressive Giant Cell Tumor of Bone With Pulmonary Distant Metastasis and Extrapulmonary Seeding in Pregnancy

**DOI:** 10.5435/JAAOSGlobal-D-19-00161

**Published:** 2020-01-09

**Authors:** Khaled A. Murshed, Ahmed Mounir Elsayed, Lajos Szabados, Sameera Rashid, Adham Ammar

**Affiliations:** From the Department of Laboratory Medicine and Pathology (Dr. Murshed, Dr. Rashid, and Dr. Ammar), the Department of Orthopedic and Musculoskeletal Oncology Surgery (Dr. Elsayed), and the Department of Radiology (Dr. Szabados), Hamad Medical Corporation, Doha, Qatar, and the Weill Cornell Medical College–Qatar (WCMC-Q), Ar-Rayyan, Qatar (Dr. Elsayed).

## Abstract

Giant cell tumor of bone (GCTB) is a locally aggressive benign neoplasm that is associated with a large biological spectrum ranging from latent benign to highly recurrent and occasionally metastatic tumor. In this article, we present a case of a 26-year-old woman who presented with swelling at the left lower ribs during pregnancy. Surgical excision was done, and histopathology showed tumor with features consistent with GCTB. MRI preformed after delivery revealed recurrence of the mass with extensive growth reaching 17 cm with two subcutaneous satellite nodules in the adjacent abdominal wall. positron emission tomography-computed tomography (PET-CT) scan revealed bilateral fluorodeoxyglucose (FDG)-avid lung nodules. Surgical resection was done, and histopathology showed no evidence of malignant transformation. Few months later, the tumor recurred again, with peritoneal deposits. The patient underwent wide massive resection of the recurrent mass and then started on denosumab therapy. Molecular analysis of the tumor detected H3F3A G34W mutation with no copy number alterations. We are presenting this case of GCTB with pulmonary distant metastasis and extrapulmonary seeding to upsurge awareness among clinicians about the possible extreme aggressive biological behavior of GCTB that can mimic the presentation of malignant bone tumor and also to discuss the possible predictive factors of such aggressive behavior.

Giant cell tumor of bone (GCTB) is a benign locally aggressive bone tumor that has a capacity to metastasize. It accounts for approximately 5% of all primary bone tumors.^1^ It is slightly more common in females and occurs most commonly in ages between 20 and 40 years. The tumor arises most commonly from the epiphysis of long bones. It has wide biological spectrum ranging from latent benign to highly recurrent and occasionally metastatic bone tumor.^2^ Metastasis occurs in approximately 2% to 5% of cases, most commonly to the lungs.^1,3^ In this article, we present an unusual case of conventional GCTB with an aggressive clinical course mimicking the behavior of malignant bone tumor. The tumor underwent rapid progression and growth during pregnancy, exhibited aggressive behavior in the form of multiple recurrences with cutaneous and peritoneal seeding along with distant metastasis to the lungs.

## Case Presentation

A 26-year-old Sudanese woman presented to a private hospital with painless swelling at the left lower ribs for 6 months' duration that showed rapid increase in size over the last 3 weeks before her presentation. She was 16 weeks pregnant at that time. Ultrasonography showed a well-defined complex mass at the left anterolateral costal margin measuring 2 × 1 × 1.4 cm. Fine needle aspiration done at the private institute showed atypical cells, followed by an unplanned excision done under local anesthesia 1 month later.

Review of the paraffin blocks and hematoxylin and eosin–stained slides at the pathology department of our facility revealed multiple fragments of tumor composed of mononuclear stromal cells with abundant large osteoclast-type multinucleated giant cells. The tumor was extending to the adjacent soft tissue and skeletal muscle. There was no evidence of marked atypia, necrosis, or atypical mitotic figures. The morphological and radiological features were consistent with GCTB. The patient was then referred to our facility for more advanced care.

Two months after the initial procedure, clinical follow-up revealed reappearance of soft-tissue mass at the site of surgery. Ultrasonographic examination confirmed the presence of a heterogeneous lesion measuring 7.4 × 4.2 cm at the site of surgery involving the left 11th rib with increased vascularity on Doppler examination. The decision by the bone tumor multidisciplinary team was to closely follow the patient up clinically, with imaging studies to be postponed after delivery of the baby. After the birth of her child, CT and MRI of the thorax were done and showed a large heterogeneous soft-tissue mass measuring 17 × 12 × 8 cm in the left side of the chest arising from and destructing the 11th rib with intra-abdominal extension to the left side of the peritoneum, compressing the lower half of left kidney and displacing the bowel loop medially. Two small nodules were seen in the adjacent abdominal wall measuring 8 mm and 12 mm. The overall picture was suggestive of a local recurrence of the tumor which was confirmed by histopathologic examination of the ultrasound-guided biopsy taken from the lesion.

**Figure 1:**
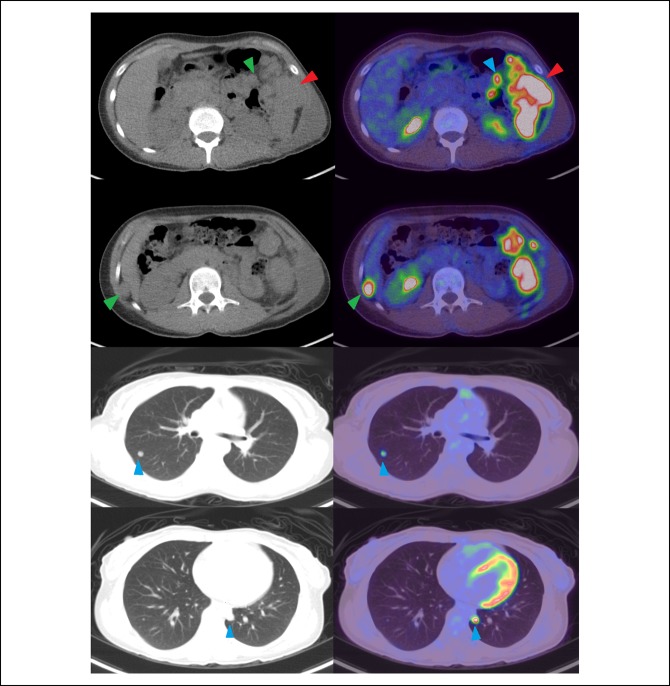
CT (left) and fused (right) FDG-PET CT images showing the large irregular left abdominal wall soft-tissue mass with intense FDG-uptake (red arrowheads), peritoneal lesions (green arrowheads), and FDG-avid bilateral pulmonary nodules (blue arrowheads).

**Figure 2:**
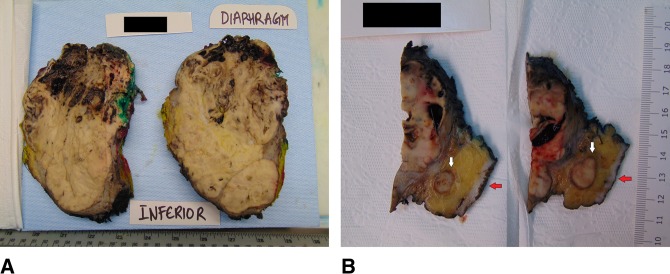
**A**, The tumor is bulky and has heterogeneous white, yellow tan focally hemorrhagic cut surfaces. **B**, Two subcutaneous tumor deposits (white arrows) are identified near the tumor beneath the skin surface (red arrows).

**Figure 3:**
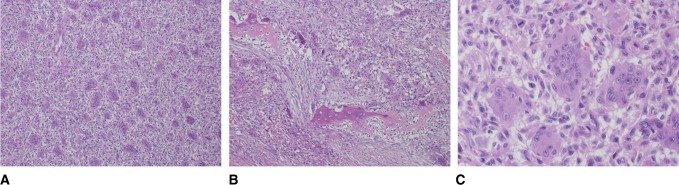
**A**, Photomicrograph depicting a tumor composed of mononuclear stromal cells admixed with osteoclast-type multinucleated giant cells (hematoxylin and eosin [H&E] stain ×100). **B**, The tumor is destructing pre-existing bony trabeculae (H&E stain ×100). **C**, High power view shows that the mononuclear stromal tumor cells are bland with no marked nuclear atypia. A mitotic figure appears at the upper left corner; however, there are no atypical forms (H&E stain ×400).

PET-CT scan examination confirmed the presence of hypermetabolic large mass centered on the left lower chest wall along with peritoneal involvement and bilateral FDG-avid lung nodules consistent with lung involvement (Figure 1). Complete excision of the recurrent mass was done with excision of the anterior parts of the 10th and 11th left ribs and releasing the tumor from the inferior surface of the left side of the diaphragm and the peritoneum. The tumor was ruptured during its release from the inferior surface of the left diaphragmatic copula. Repair of the diaphragm was done with mesh reconstruction of the defect at the left upper anterior abdominal wall. Histopathologic gross examination of the resected specimen showed that the tumor had heterogonous white, yellow to brown, and focally hemorrhagic cut surfaces with two subcutaneous skin nodules found in the vicinity of the tumor (Figure 2, A and B). Microscopically, the tumor showed morphological features similar to the initial tumor (Figure 3, A and B). No marked cytological atypia, atypical mitosis, tumor necrosis, or any other features suggestive of malignant transformation were noted (Figure 3, C).

Four months after this procedure, another recurrence of the tumor occurred. The patient started to feel pain at the surgical site radiating to the lower abdomen. Examination revealed palpable mobile hard mass at the surgical bed. MRI confirmed the presence of multiple variable sized large intra-abdominal and retroperitoneal soft-tissue lesions, denoting recurrence of the tumor.

PET-CT scan confirmed the presence of multiple peritoneal and abdominal wall lesions on the left with perihepatic peritoneal lesion on the right as well. The previously seen lung involvements showed increasing size and uptake. Ultrasound-guided biopsy from the recurrent lesion was done to rule any malignant transformation. However, no features of malignancy were noted.

Considering the atypical and very aggressive clinical behavior of the tumor, it was sent for DNA methylation study. Molecular testing was done on genomic DNA extracted from the tumor in formalin-fixed paraffin embedded tissue to detect hotspot mutations in the H3.1 gene (Hist1H3A and Hist1H3B). H3F3A G34W mutation was detected, but no copy number alterations were found.

The patient had another pregnancy, and rapid progression of the tumor was noticed. She travelled to another medical facility where she had termination of her pregnancy followed by wide massive resection of the left retroperitoneal local recurrence together with partial resection of left diaphragmatic copula, left-sided hemicolectomy with end-to-end transversosegmoidostomy, and extensive resection of the abdominal wall with mesh reconstruction.

Clinical and radiological follow-up of the patient (6 months after the latest procedure) showed no evidence of local recurrence and a stable course of the lung nodules. The patient was on denosumab for 3 months after the last surgery, and the latest PET-CT scan did not reveal any recurrence or change in the size of lung nodules. She is now on regular follow-up.

## Discussion

GCTB is a locally aggressive primary bone neoplasm that accounts for about 5% of all primary bone tumors.^1^ It is slightly more common in females and occurs most commonly in ages between 20 and 40 years. It has a major preference for the epiphysis of the long bones (distal femur, proximal tibia, distal radius, and proximal humerus). The tumor is locally aggressive with high rate of local recurrence. Distant metastasis can occur in up to 5% of cases, most commonly to the lungs.^1,3^ There are isolated case reports of metastasis to other sites such as lymph nodes, bone, skin, and breast.^4,5,6,7,8^

The tumor is characterized morphologically by proliferation of mononuclear stromal cells which comprise the neoplastic component of the tumor admixed with numerous osteoclast-like multinucleated giant cells. The multinucleated giant cells can be very large with up to 100 nuclei and have even distribution. A variable number of mitotic figures can be seen; however, the presence of atypical forms suggests malignancy.

Our case is unusual in many aspects. First, the patient was diagnosed with GCTB for the first time during pregnancy, when she presented with a mass arising from the left 11th rib. The tumor was initially small measuring 2 cm in maximum dimension. Unplanned excision under local anesthesia was done. Two months later, clinical examination revealed reappearance of soft-tissue mass at the site of surgery, and ultrasonographic examination confirmed the presence of a mass measuring 7.4 × 4.2 cm. This is mostly a progressively growing residual tumor that was not completely excised at the initial procedure especially that the procedure was done under local anesthesia. MRI done after delivery revealed a huge mass measuring 17 cm in maximum dimension at the same location. The rapid progression of GCTB in pregnancy is well established in the literature.^9,10^ Kim et al^10^ reported a rare case of GCTB involving the metacarpal bone with rapid growth during pregnancy. We have done immunohistochemical stains for hormonal receptors including estrogen receptor and progesterone receptor, which may explain the rapid growth of these tumors in pregnancy if they are overexpressed. Estrogen receptor was negative, but progesterone receptor showed focal weak nuclear staining in our case.

Second, the patient also developed bilateral multiple lung nodules demonstrated by PET-CT scan that was done after delivery. The nodules have radiological features consistent with metastatic GCTB. Metastasis occurs rarely in GCTB, and it most commonly develops in the lungs. The factors that contribute to increase risk of metastasis in GCTB may include the primary site of the tumor, history of multiple local recurrences, and modality of treatment of the primary tumor.^8^ The tumor in our case was arising from the left 11th rib. Pulmonary metastasis was detected for the first time by PET-CT scan done after delivery after the first recurrence of the tumor.

In our experience, there are no morphological features that can predict the clinical behavior of GCTB. We did extensive sampling to rule out the possibility of malignant transformation, which can occur rarely in GCTB, and may explain the aggressive clinical course in our case. Primary malignancy in GCTB is seen at initial diagnosis as an area of high-grade sarcoma within an otherwise conventional GCTB. In secondary malignant GCTB, a high-grade sarcoma arises subsequent to previous radiation or surgical treatment, and the pre-existing GCTB is not always evident anymore.^11^ In our case, no atypical mitotic figures, marked nuclear atypia, or any features of malignant transformation were noted in all recurrences.

Moreover, our patient developed two subcutaneous skin nodules over the abdomen after the first recurrence. Histopathologic examination revealed that those nodules have the same morphological appearance of the main tumor; therefore, they are regarded as local cutaneous tumor seeding after the initial surgery. No features of malignant transformation were identified in the skin deposits as well.

The aggressive clinical behavior of the tumor in the terms of multiple local recurrences along with regional seeding and distant metastasis as well as the rapid progression encouraged us to send it for molecular analysis. H3F3A G34W mutation, which is a characteristic feature of conventional benign GCTB, was detected. This mutation can be found in more than 90% of cases of conventional GCTB.^12,13^ H3F3A mutational testing may be a useful adjunct to differentiate GCTB from giant cell-rich sarcomas. Although the presence of H3F3A mutations does not exclude with certainty a diagnosis of sarcoma, the possibility of a malignant evolution of GCTB should also be considered.^11^ No copy number alterations were detected in our case.

The treatment choices of GCTB are essentially curettage with adjuvants is reasonable depending on the extent of the soft-tissue component. If initially inoperable, neoadjuvant systemic targeted therapy may facilitate intralesional surgery at a later stage. Recently, denosumab has become a new treatment option for locally advanced GCTB. Denosumab is an inhibitor for receptor activator of nuclear factor kappa-Β ligand that blocks osteoclast maturation. It remains unknown whether local recurrence rate will be affected by denosumab treatment.^14^

In summary, we are reporting an unusual case of conventional GCTB that underwent rapid progression and growth during pregnancy, exhibited aggressive behavior in the form of multiple recurrences with regional cutaneous and peritoneal seeding and distant metastasis to lungs, mimicking the behavior of malignant bone tumor. The patient life quality was affected by the consequences of this aggressive tumor as she had to terminate her second pregnancy. There were no histological features of malignant transformation. In our experience, the histomorphological and radiological features alone cannot predict the clinical behavior of these tumors. It is not clear whether the genotype and molecular alterations of this tumor can predict the biological behavior, and further study and research should be pursued.
